# The antimicrobial peptide DGL13K is active against drug-resistant gram-negative bacteria and sub-inhibitory concentrations stimulate bacterial growth without causing resistance

**DOI:** 10.1371/journal.pone.0273504

**Published:** 2022-08-25

**Authors:** Sven-Ulrik Gorr, Hunter V. Brigman, Jadyn C. Anderson, Elizabeth B. Hirsch

**Affiliations:** 1 Department of Diagnostic and Biological Sciences, University of Minnesota School of Dentistry, Minneapolis, Minnesota, United States of America; 2 Department of Experimental and Clinical Pharmacology, University of Minnesota College of Pharmacy, Minneapolis, Minnesota, United States of America; Nanyang Technological University, SINGAPORE

## Abstract

Antimicrobial peptides may be alternatives to traditional antibiotics with reduced bacterial resistance. The antimicrobial peptide GL13K was derived from the salivary protein BPIFA2. This study determined the relative activity of the L-and D-enantiomers of GL13K to wild-type and drug-resistant strains of three gram-negative species and against *Pseudomonas aeruginosa* biofilms. DGL13K displayed in vitro activity against extended-spectrum beta-lactamase (ESBL)-producing and *Klebsiella pneumoniae* carbapenemase (KPC)-producing *Klebsiella pneumoniae* (MICs 16–32 μg/ml), MDR and XDR *P*. *aeruginosa*, and XDR *Acinetobacter baumannii* carrying metallo-beta-lactamases (MICs 8–32 μg/ml). *P*. *aeruginosa* showed low inherent resistance to DGL13K and the increased metabolic activity and growth caused by sub-MIC concentrations of GL13K peptides did not result in acquired bacterial resistance. Daily treatment for approximately two weeks did not increase the MIC of DGL13K or cause cross-resistance between LGL13K and DGL13K. These data suggest that DGL13K is a promising antimicrobial peptide candidate for further development.

## Introduction

Antimicrobial peptides (AMPs) have been considered as an alternative to traditional antibiotics and may represent a different therapeutic modality with reduced opportunity for bacterial resistance [1, 2]. The possibility of bacterial resistance to AMPs has been extensively debated. On the one hand, it has been proposed that their mode of action at the cell membrane makes resistance unlikely [1, 3, 4] and peptides such as polymyxin B and nisin have been used for decades with no significant resistance [5]. On the other hand, resistance can be generated under laboratory conditions [6, 7] causing concerns that bacteria that become resistant to a therapeutic AMP would also be resistant to endogenous human host-defense peptides (“arming the enemy”) [8–10], as shown for pexiganan and HNP-1 [6]. A recent study suggests that AMPs are more likely to show collateral sensitivity rather than cross-resistance to traditional antibiotics [2]. In addition, we have recently reported that closely related peptide enantiomers can show significant differences in their interactions with bacterial defense mechanisms [11, 12].

We previously described the design of anti-inflammatory and bacterial agglutinating peptides based on the sequence of the human salivary protein BPIFA2 [13–16]. A modified peptide, GL13K, was developed by substituting three polar or charged amino acids with lysine residues [17]. The resulting peptide is a more cationic and highly bactericidal peptide, which retains anti-inflammatory activity in vitro and in vivo [17]. A second generation, D-enantiomer of GL13K (DGL13K) resists bacterial proteases [12, 18] and is bactericidal against gram-negative and gram-positive bacteria, including vancomycin-resistant *Enterococcus faecalis* and methicillin-resistant *Staphylococcus aureus* [12, 19]. Interestingly, a similar D-enantiomer selectivity of gram-positive bacteria was reported for the AMP M33-D [20]. The goal of this study was to determine the relative activity of the L-and D-enantiomers of GL13K to wild-type and drug-resistant strains of gram-negative bacteria and bacterial biofilms. In addition, we show that *Pseudomonas aeruginosa* exhibit hormesis in response to subinhibitory concentrations of the GL13K peptides but this does not result in acquired resistance to DGL13K or cross-resistance between the L- and D-enantiomers of GL13K.

## Materials and methods

### Bacterial isolate collection

The laboratory strains, *P*. *aeruginosa* Xen41, a bioluminescent derivate of PA01 (Xenogen, Alameda, CA; now Perkin-Elmer, Waltham, MA), *P*. *aeruginosa* ATCC 27853, and *Klebsiella pneumoniae* ATCC 13883 were used as quality control strains and analyzed in parallel with each minimal inhibitory concentration (MIC) experiment. Four clinical *P*. *aeruginosa* strains (55, 147, 220, 237) collected from Boston, MA, and 2 clinical strains (507, 508) from Philadelphia, PA were tested [21]. Six clinical isolates of *K*. *pneumoniae* were tested including three from Boston, MA (19, 127, 132) and three from Philadelphia, PA (556, 584, 596). Finally, six *Acinetobacter baumannii* isolates acquired from the Gram Negative Carbapenemase Detection and *A*. *baumannii* panels of the CDC & FDA Antibiotic Resistance Isolate Bank (Atlanta, GA) (http://www.cdc.gov/arisolatebank) were tested: AR Bank #33, #52, #102, #280, #290, and #294. The resistance phenotype for each strain is listed in **Table 1**. Isolates were characterized as multidrug-resistant (MDR) if non-susceptible to ≥1 agent in ≥ 3 antimicrobial categories, and extensively-drug resistant (XDR) if non-susceptible to ≥ 1 agent in ≥ 6 antimicrobial categories [22].

**Table 1:** LGL13K and DGL13K MIC values determined against wild-type and drug-resistant strains of gram-negative bacteria.

Species	Strain	Clinical isolate reference	Resistance phenotype	Resistance mechanisms	MIC: LGL13K (μg/ml)	MIC: DGL13K (μg/ml)
*P*. *aeruginosa*	PA01	N/A	Reference		128	32
	ATCC27853	N/A	Reference ATCC		128	32
	55	[21]	XDR phenotype	ND	128	64
	147	[21]	MDR phenotype	ND	128	64
	220	[21]	Wild-type	ND	128	64
	237	[21]	MDR phenotype	ND	64	32
	507	Present study	MDR phenotype	ND	>128	128
	508	Present study	MDR phenotype	ND	128	64
*A*. *baumannii*	AR Bank #33	N/A	XDR phenotype	NDM-1, OXA-94, sul2	64	16
	AR Bank #52	N/A	XDR phenotype	OXA-100, OXA-58, sul2	64	8
	AR Bank #102	N/A	XDR phenotype	ADC-25, armA, catB8, mph(E), msr(E), OXA-66, strA, strB, sul1	128	32
	AR Bank #280	N/A	XDR phenotype	aac(3)-Ia, ADC-25, aph(3’)-Ic, OXA-66, strA, strB, sul1, TEM-1D	64	32
	AR Bank #290	N/A	XDR phenotype	ADC-25, aph(3’)-Ic, aph(3’)-VIa, armA, catB8, mph(E), msr(E), OXA-23, OXA-66, strA, strB, sul1, TEM-1D	64	32
	AR Bank #294	N/A	XDR phenotype	aac(3)-IIa, aph(3’)-VIa, OXA-23, OXA-65, strA, strB, sul2, TEM-1B	64	32
*K*. *pneumoniae*	ATCC 13883	N/A	Reference ATCC		64	8
	19	[21]	Wild-type	ND	64	16
	127	[21]	Wild-type	ND	64	16
	132	[21]	ESBL phenotype	ND	64	32
	556	[25]	Carbapenem-resistant MDR phenotype	KPC-2	64	16
	584	[25]	Carbapenem-resistant MDR phenotype	KPC-3	64	16
	596	Present study	ESBL phenotype	ND	64	16

ESBL: extended-spectrum beta-lactamase; KPC: *Klebsiella pneumoniae* carbapenemase; MDR: multidrug-resistant; N/A: not applicable; ND: not determined; XDR: extensively drug-resistant

### Peptides

Polymyxin B was purchased from MilliporeSigma (St. Louis, MO). LGL13K (GKIIKLKASLKLL-NH2) [17] and an all-D-amino acid version of this peptide (DGL13K) [12, 18] were purchased from Bachem AG (Bubendorf, Switzerland). The non-bactericidal control peptide GL13NH2 (GQIINLKASLDLL-NH2) [16, 17] was purchased from Aapptec (Louisville, KY). GL13K peptides were synthesized by Fmoc chemistry and the TCA form isolated at >95% purity by reverse-phase HPLC. The purity and identity of each peptide were verified by the suppliers by reverse-phase HPLC and mass spectrometry, respectively. The lyophilized peptides were re-suspended in 0.01% sterile acetic acid at 10 mg/ml and stored at 4°C. All peptide batches were validated by MIC testing prior to use, using the modified Hancock protocol described below.

### MIC determinations

MICs were determined via two different methods: the broth microdilution reference method used for traditional antibiotic susceptibility testing as recommended by the Clinical and Laboratory Standards Institute [23] and the Modified Hancock protocol, a broth microdilution method for cationic AMPs [24].

#### CLSI broth microdilution protocol

MICs were determined in at least duplicate on separate days. *P*. *aeruginosa* ATCC 27853 was used as a control strain and analyzed in parallel with each experiment. Briefly, all test isolates and ATCC reference strains were subcultured twice consecutively onto blood agar plates from storage at -80°C and incubated overnight at 35°C. Single isolated colonies were used to inoculate cation-adjusted Mueller-Hinton broth (BBL, Becton Dickinson and Company, Sparks, MD) to a final density of approximately 5 × 10^5^ colony-forming units (CFU)/ml in each well of a 96-well plate. Bacterial inocula were verified via enumeration following plating of ten-fold dilutions of the inoculum suspension.

#### Modified Hancock protocol

Broth microdilution assay for cationic antimicrobial peptides [24] was performed as previously described [19]. Briefly, a 20 μl solution (1 mg/ml) of each peptide was serially diluted 2-fold in a 1:10 dilution of phosphate-buffered saline (PBS) (Hyclone; GE Healthcare, Pittsburgh, PA) in dH2O (10%PBS), and then mixed with 100 μl of *P*. *aeruginosa* Xen 41 (10^5^ CFU/ml) in Mueller-Hinton Broth. Final volume in each well was 120 μl and the peptide concentration range tested was 167 μg/ml– 0 μg/ml. Samples were incubated in polypropylene plates at 37°C overnight with gentle shaking. The optical density at 630 nm (OD630) and luminescence were read in a Synergy HT plate reader (BioTek, Winooski, VT) and plotted against peptide concentration. The MIC was read as the lowest peptide concentration that prevented bacterial growth.

### Biofilm assay

*P*. *aeruginosa* Xen 41 (5 x 10^5^ CFU/well, 100 μl Luria-Bertani (LB) broth) were incubated with shaking overnight in 96-well microtiter plates at 37°C. The wells were aspirated and the attached biofilms washed with 200 μl PBS. To each well was added 150 μl Mueller-Hinton Broth or PBS containing a 2-fold serial dilution of peptide (concentration range 1 mg/ml– 1.95 μg/ml). The plates were incubated 60 min at 37°C and luminescence determined in a BioTek plate reader to quantify live cells.

To determine total cells (live+dead) in the attached biofilm, the wells were aspirated and washed with 2 x 200 μl PBS. The plates were incubated with 150 μl/well of 0.03% crystal violet for 30 min at room temperature. The wells were aspirated and washed with 2 x 300 μl PBS followed by 2 x 300 μl dH2O. To each well was added 200 μl 95% ethanol, incubated for 30 min at 37°C, and the OD630 determined. The readings for each peptide were normalized by dividing with the luminescence or OD of the samples with the lowest peptide concentration.

### Hormesis

To determine the effect of subinhibitory concentrations of GL13K peptides on bacterial growth and metabolic activity, MIC values (modified Hancock protocol) were read spectrophotometrically and the OD630 (growth) and luminescence (metabolic activity) were determined at each peptide concentration. The peptide concentrations were converted to fold-MIC for each peptide and plotted to allow direct comparison of peptides with different MICs.

### Frequency of resistance

LE agarose (BioExpress, Kaysville, UT) was dissolved at 1% in Mueller-Hinton Broth at 95°C. The agarose broth was cooled to 60°C, 100 μg/ml DGL13K was added and the DGL13K-agarose poured in 10 cm petri dishes. Overnight cultures of *P*. *aeruginosa* Xen41 were pelleted and suspended in sterile 0.9% saline at 5 x 10^8^ CFU/ml (an aliquot was diluted and cultured on agar to validate the concentration of the culture). One ml bacterial culture was plated on each of duplicate DGL13K-agarose plates and incubated overnight at 37°C. Surviving colonies were enumerated as a fraction of 10^9^ plated CFU.

### Serial MIC assay

This assay was performed to determine potential development of resistance, as described previously [12]. Briefly, an initial MIC assay was prepared using the modified Hancock protocol. The MIC was recorded the following day and the bacteria in the wells containing 0.5xMIC of each peptide (i.e. the highest peptide concentration that allowed growth) were diluted 1000-fold in Mueller-Hinton Broth and 100 μl/well used to inoculate a new MIC plate. The MIC assay was repeated daily for 16 days. On day 15, bacteria that had been exposed to LGL13K were treated with DGL13K to determine cross-resistance.

## Results

### Activity against drug-resistant bacteria

Drug-resistant bacteria are an increasing problem and novel antibiotics are urgently needed. The second generation AMP DGL13K has been found to be highly effective against vancomycin-resistant *E*. *faecalis* and methicillin-resistant *S*. *aureus* [12, 19]. In this study, we tested the L- and D-enantiomers of GL13K against drug-resistant strains of the gram-negative bacteria *K*. *pneumoniae*, *A*. *baumannii* and *P*. *aeruginosa* (Table 1).

**Fig 1:**
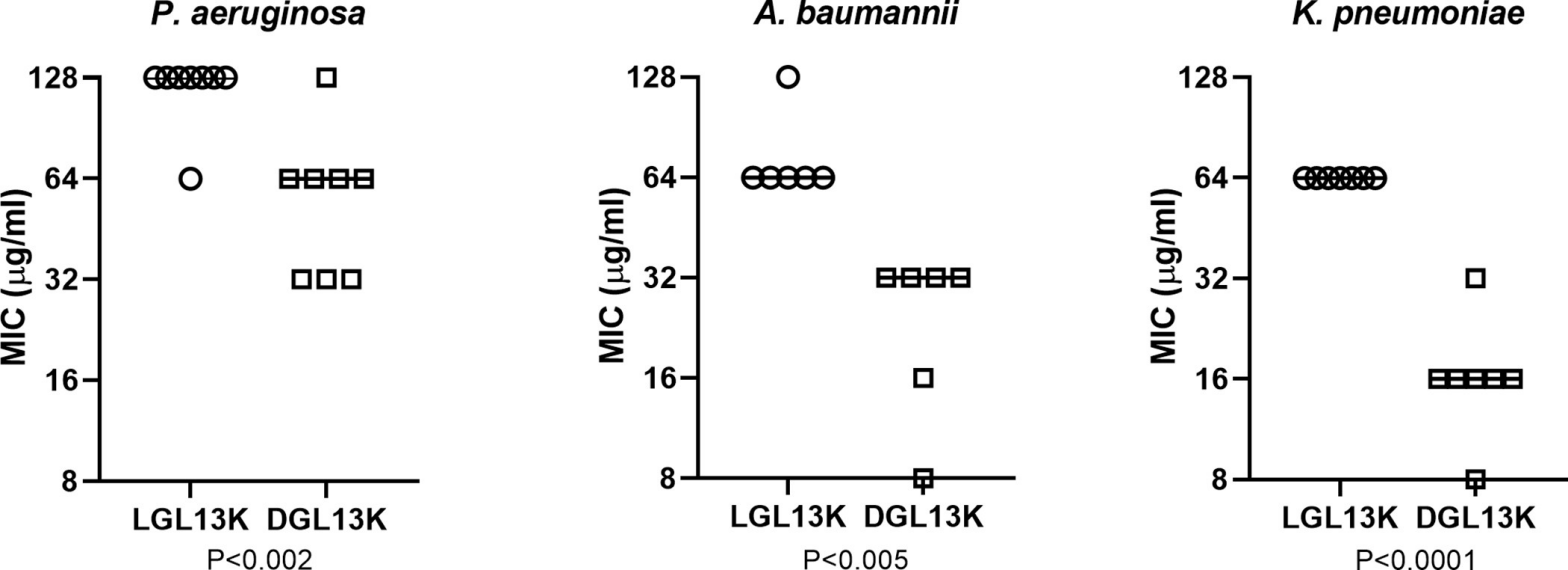
Comparison of MIC values for different strains of *P*. *aeruginosa*, *A*. *baumannii* and *K*. *pneumoniae* treated with LGL13K or DGL13K. For each species listed in Table 1, the MIC of the two peptide enantiomers were compared by paired student’s t-test. P-values are indicated. Lines represent the median MIC of each group.

The MIC for each peptide was relatively consistent between strains of each bacterial species (**Table** 1). Comparison of the MICs recorded for DGL13K and LGL13K showed that the MICs for DGL13K were generally 2-fold lower than those recorded for LGL13K (Fig 1), in agreement with our previous results [19]. Against the three gram-negative species tested, DGL13K was most active against *K*. *pneumoniae* and *A*. *baumannii*, with MICs ranging from 8–32 μg/ml (**Table** 1). Against *K*. *pneumoniae* isolates, MICs for ESBL- and KPC-producing strains did not significantly differ when compared to the ATCC reference strain. Against *P*. *aeruginosa*, MICs were within 2 doubling dilutions for MDR and XDR isolates (32–128 μg/ml) when compared to the reference strains (32 μg/ml). The latter results are about 6-fold higher than those previously reported for *P*. *aeruginosa* [19].

### Activity against biofilms of *P*. *aeruginosa*

**Fig 2:**
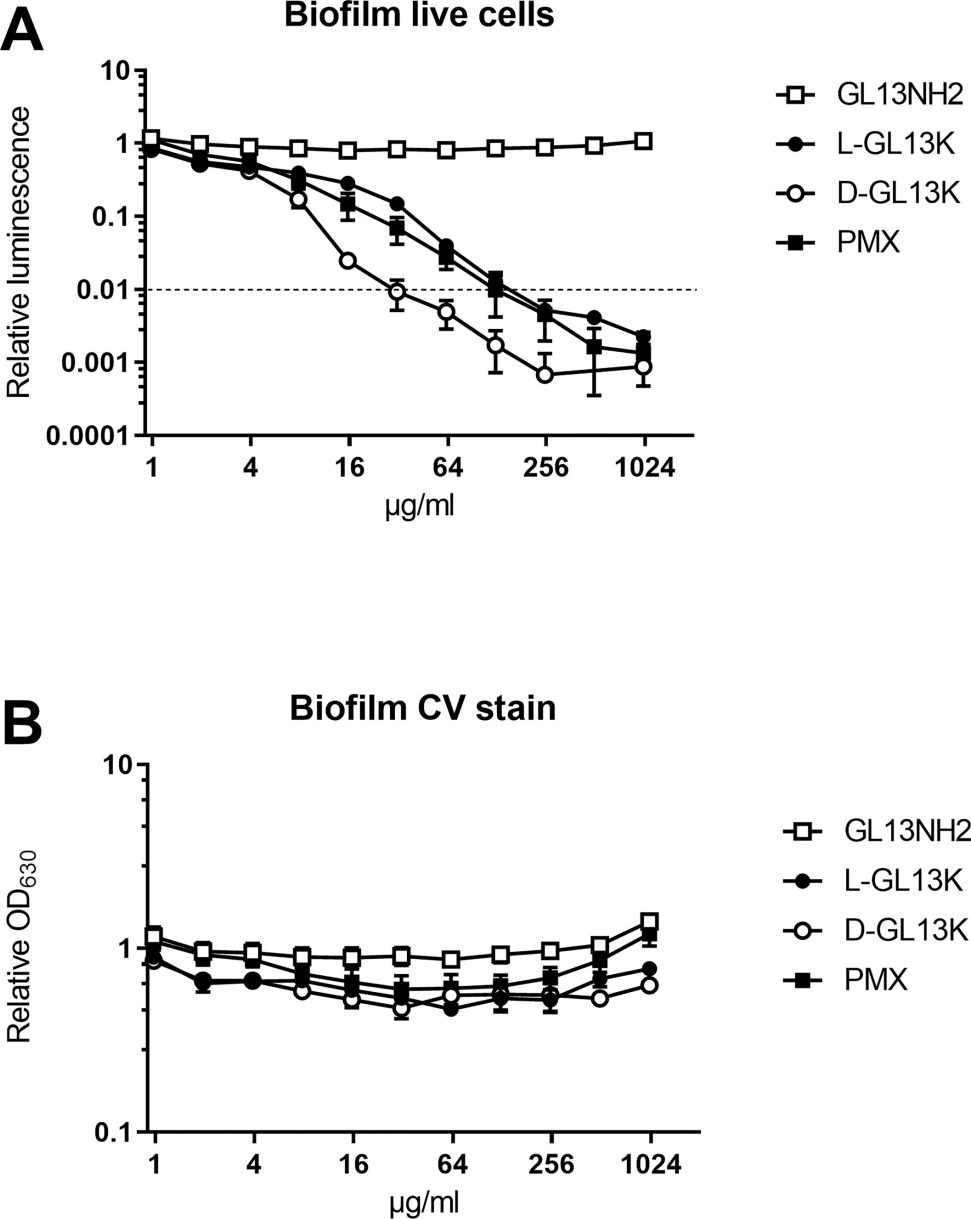
Bactericidal activity of peptides against *P*. *aeruginosa* biofilms. Biofilms were incubated for 1h with peptides, at the concentrations shown. **A.** live cells were quantitated by luminescence. Dotted line indicates 99% killing of biofilm. **B.** Biofilm biomass was quantitated by crystal violet staining. PMX = polymyxin B. Data from two independent experiments performed in duplicate were normalized to the mean of the lowest peptide concentration in each experiment. Data shown as mean ± SEM (N = 4).

LGL13K and DGL13K kill biofilms of *P*. *aeruginosa* [18]. To compare the dose needed to kill biofilms with the MIC, biofilms were incubated with increasing doses of LGL13K, DGL13K, GL13NH2 and the control antimicrobial peptide polymyxin B. Fig 2A shows that 99% reduced viability (LD99) of wild-type *P*. *aeruginosa* was achieved at a concentration of 32 μg/ml, whereas 128 μg/ml of LGL13K or polymyxin B were required to reach a comparable reduction of viability. Thus, the LD99 for biofilms is similar to the MIC achieved for both GL13K enantiomers (Table 1). Biofilm viability was not affected by the control peptide GL13NH2, which is not bactericidal [17]. The biomass of the biofilms was not reduced by the peptide treatments, as evidenced by crystal violet staining of attached bacteria (Fig 2B). It has previously been demonstrated that killed biofilm can remain attached on DGL13K-coated surfaces [26].

### Effect of sub-inhibitory peptide concentrations on bacterial growth

**Fig 3:**
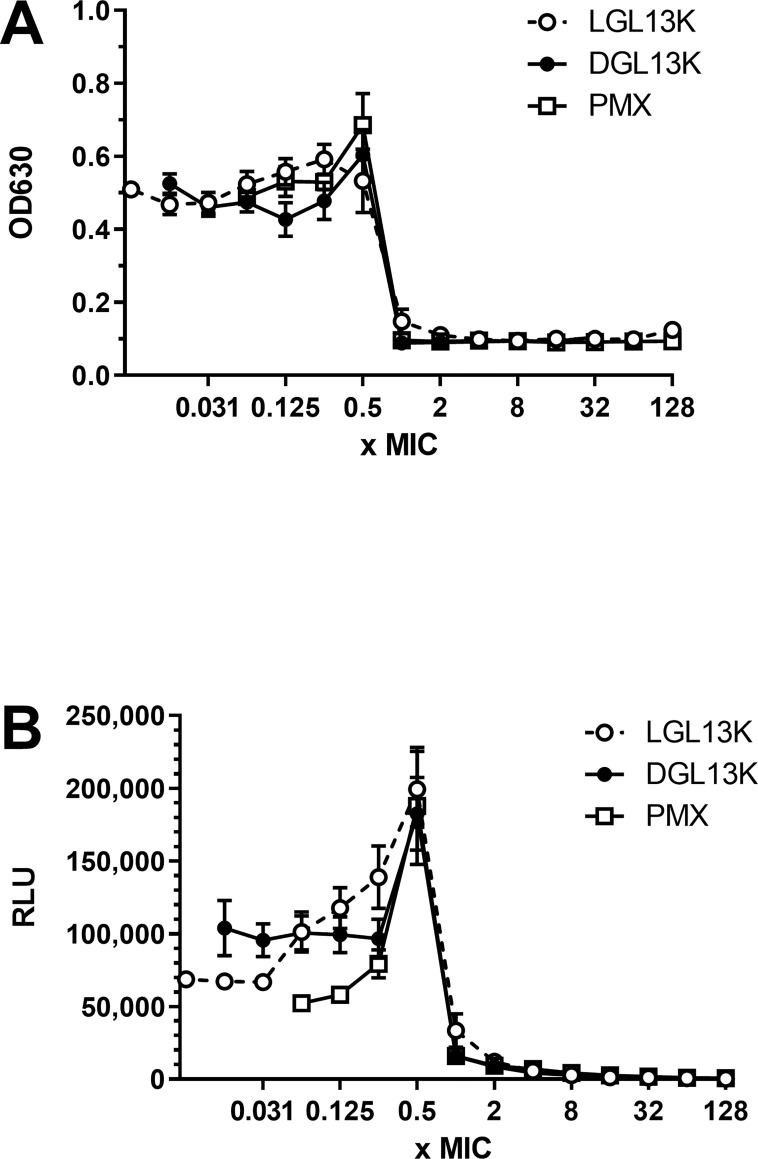
*P*. *aeruginosa* culture response to increasing peptide concentrations. *P*. *aeruginosa* Xen 41 were incubated with increasing concentrations of LGL13K (open circles), DGL13K (closed circles) or polymyxin B (PMX, open squares). OD at 630 nm (A) and luminescence (RLU) (B) were recorded at peptide concentrations corresponding to 0.007 to 128x MIC for each peptide. The data from 2–3 independent experiments are shown as mean ± SEM, N = 4–8.

In dose response experiments with *P*. *aeruginosa*, we noted that the OD_630_ of the cultures increased with increasing peptide concentration, up to 0.5 x MIC. A similar effect of subinhibitory concentrations has been reported for many toxic substances (hormesis), including traditional antibiotics [27]. To further evaluate this effect, the growth of *P*. *aeruginosa* was determined by culture density (OD_630_) and bacterial metabolic activity was determined by cellular luminescence [28]. Increasing peptide concentration up to 0.5 x MIC increased the OD_630_ by about 50% (Fig 3A) while metabolic activity increased 2-4-fold (Fig 3B).

### Bacterial resistance to L- and D-GL13K

The frequency of resistance of *P*. *aeruginosa* was less than 10^**−9**^ when the bacteria were plated on agar containing 100 μg/ml DGL13K, i.e. 3xMIC determined in **Table 1**. Thus, these bacteria show very low inherent resistance to DGL13K.

**Fig 4:**
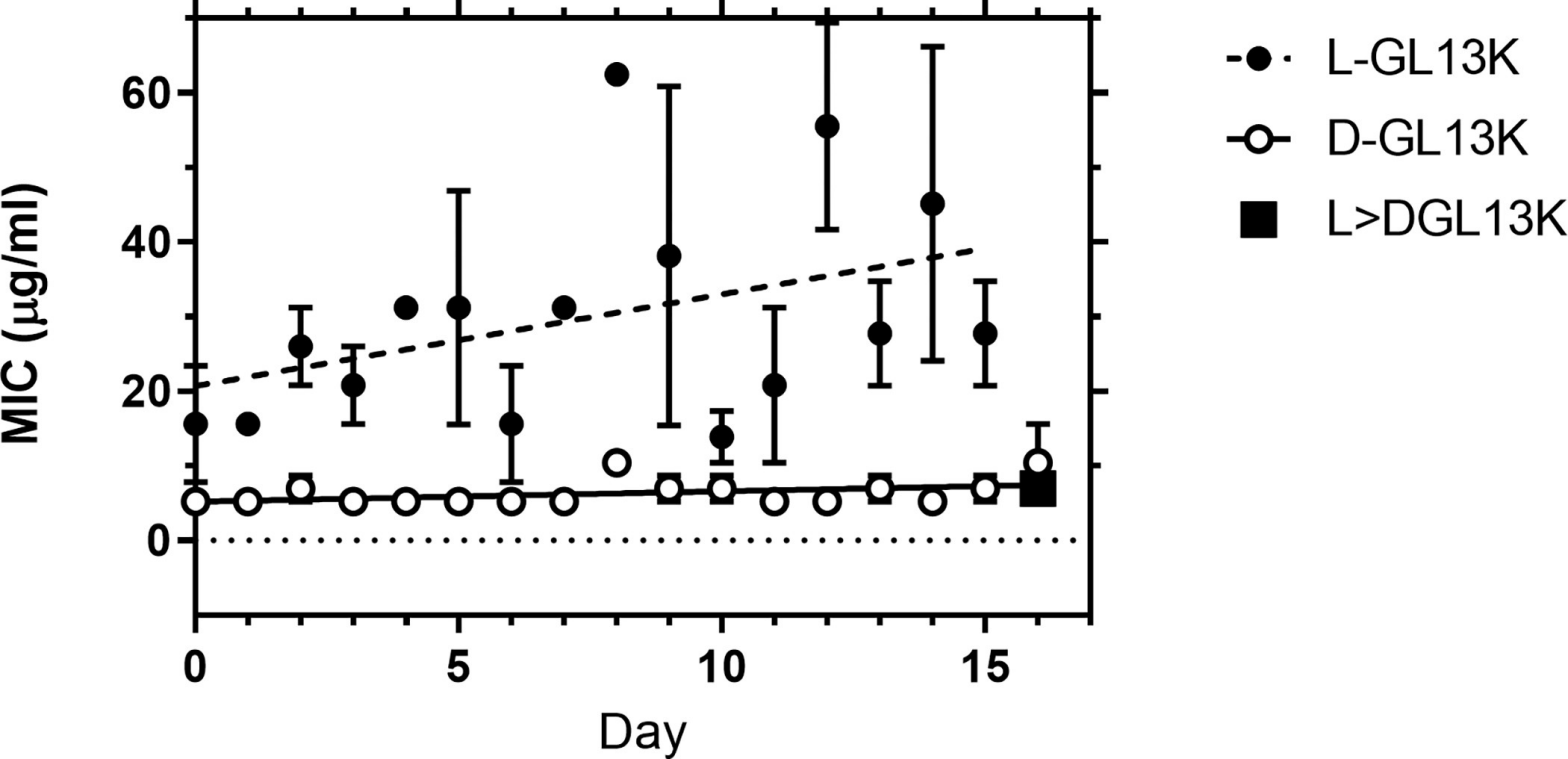
Development of resistance in *P*. *aeruginosa* by repeated treatment with LGL13K (closed circles) or DGL13K (open circles). The MIC determinations were plotted for each day (mean ± SEM, N = 3) and analyzed by linear regression. Samples treated with LGL13K for 15 days were then treated with DGL13K and the MIC determined (square. Mean ± SEM, N = 3).

The increased metabolic activity caused by culturing *P*. *aeruginosa* in the presence of 0.5xMIC of the GL13K peptides raised the question if the bacteria acquire resistance to the GL13K enantiomers when they are cultured under sub-inhibitory peptide concentrations. Repeated exposure of *P*. *aeruginosa* to 0.5xMIC of DGL13K did not increase the MIC of this peptide after 16 rounds (days) of selection (Fig 4). The MIC for LGL13K trended towards a 2-fold increase but this did not reach statistical significance (P<0.06). Importantly, bacteria that had reached the higher MIC for LGL13K did not show an increased MIC for DGL13K (Fig 4, closed square). The lack of cross-resistance between these two closely related AMP enantiomers is promising for future clinical use [10].

## Discussion

The antimicrobial peptide enantiomers LGL13K and DGL13K have shown promising activity against gram-negative (LGL13K and DGL13K) [17, 18] and gram-positive bacteria (DGL13K) [12, 19]. In this report, we determined the antibacterial activity against additional bacterial species as well as several drug-resistant strains of clinically important gram-negative bacteria. The second-generation antimicrobial peptide DGL13K shows activity against the tested drug-resistant strains that is similar to that of the corresponding wild-type strains. Importantly, DGL13K displayed activity against isolates of multiple species of resistant gram-negative pathogens including ESBL and KPC-producing *K*. *pneumoniae* (MICs 16–32 μg/ml), MDR and XDR *P*. *aeruginosa*, and XDR *A*. *baumannii* carrying metallo-beta-lactamases (MICs 8–32 μg/ml). Treatment options for infections caused by these resistant pathogens are limited and often result in treatment with more toxic agents such as the polymyxins or aminoglycosides [29, 30].

The MICs for DGL13K were consistently about 2-fold lower than the corresponding value for LGL13K (Fig 1). This is consistent with our earlier finding that LGL13K, but not DGL13K, is inactivated by conditioned bacterial medium while the activity is preserved in the presence of EDTA [18]. These results suggested that a bacterial metalloprotease is involved in the degradation of LGL13K in *P*. *aeruginosa* [18]. Similarly, we have reported that *Enterococcus faecalis* protease can degrade LGL13K, but not DGL13K [12]. Thus, in an overnight MIC assay there is increasing bacterial growth and release of proteases that would increase the apparent MIC for the susceptible LGL13K peptide.

The circular dichroism and NMR structures of LGL13K have been resolved in phospholipid environments that mimicked those of bacterial membranes [31, 32]. Once the peptide reaches the cell membrane, it transitions from random coil, through α-helix, to a ß-sheet structure that presumably represents the active conformation. The ß-sheet structure aligns with model membranes and has a high capacity to disrupt membrane order [31]. Analysis of DGL13K secondary structure suggests that its interaction with LPS-rich (Gram negative-like) membranes is similar to that of LGL13K [32]. In this context the negative charge of the target membrane attracts the higher concentrations of the cationic peptide needed for ß-sheet formation and membrane perturbation [31].

DGL13K did not exhibit collateral sensitivity [2] in drug-resistant strains of gram-negative bacteria. Similarly, we have recently reported that the MIC for drug-resistant *Staphylococcus aureus* and *Enterococcus faecalis* are not lower than the MIC for wild-type strains [12, 19]. Indeed, bactericidal activity is similar to the growth inhibiting activity in most cases and the peptide is highly active against established bacterial biofilms. Thus, DGL13K is a promising candidate for further development.

The data in Table 1 were generated using the CLSI protocol for broth microdilution [23] while our previous results [17–19] were obtained with a modified version of the protocol developed for cationic antimicrobial peptides by Hancock [24]. Similarly, it has been reported that the ‘Hancock protocol’ results in lower MICs for cationic peptides than the protocol described by CLSI [33]. The exact cause of the higher MIC values for the CLSI protocol is not clear. However, the CLSI protocol is not optimized for antimicrobial peptides and the different results compared to the Hancock protocol likely result from a combination of factors embedded in the two protocols.

In the course of antibacterial activity studies, we noted that subinhibitory (sub-MIC) concentrations of the antimicrobial peptides caused increased growth and metabolic activity of *P*. *aeruginosa*, which was most notable at 0.5xMIC. A similar phenomenon (hormesis) has been described for several toxins in multiple species and taxa [34, 35]. Interestingly, the effect in bacteria has been linked to “medium-richness”. Thus, the growth promoting effect of the antibiotics sulfamethazine and erythromycin were more pronounced in dilute Mueller-Hinton Broth than in full strength Mueller-Hinton Broth and the effect was absent in Luria-Bertani medium [27]. The effect of GL13K peptides was stronger on metabolic activity than overall growth, suggesting that *P*. *aeruginosa* were selectively stimulated in metabolic pathways. Consistent with this observation, hormesis has been suggested to constitute a defensive adaptation to low concentrations of stressors that affects the transcriptional activity of bacteria [36]. Thus, it is likely that low concentrations of antibiotics cause an adaptive response that increases survival upon further increases in antibiotic concentration.

Re-analysis of peptide dose-response curves (MIC assays) for the gram-positive bacteria *Streptococcus gordonii*, *Enterococcus faecalis* [12] and *Staphylococcus aureus* [19] revealed that some strains exhibited a similar growth stimulatory effect at subinhibitory concentrations of GL13K peptides (Gorr, unpublished). DGL13K, but not LGL13K, circumvents cell wall defense mechanisms that include D-alanylation of teichoic acids. Interestingly, the hormesis effect was observed in D-alanylation mutants that had lost resistance to LGL13K, suggesting that the effect is not associated with the initial point of attack at the cell surface. A better understanding of the cellular targets for GL13K peptides in gram-negative and gram-positive bacteria will be needed to determine the different mechanisms that allow peptide-induced bacterial hormesis.

*P*. *aeruginosa* show very low inherent resistance to DGL13K and the increased metabolic activity and growth at sub-MIC concentrations of GL13K peptides did not result in acquired bacterial resistance. Daily treatment for about two weeks had no effect on the MIC of DGL13K towards *P*. *aeruginosa*. Similar results were recently reported for the gram-positive bacteria *S*. *gordonii* and *E*. *faecalis* treated with DGL13K [12]. A likely explanation is that upregulation of bacterial proteases is an effective defense against the L-enantiomer of antimicrobial peptides. This readily explains the increased MIC observed for LGL13K (Fig 1) and the lack of resistance to DGL13K (Fig 4). The proposed translational response to sub-inhibitory doses of peptide (hormesis) [36] further supports that a simple upregulation of bacterial proteases can increase cellular resistance. As a further resistance mechanism, surface modification has also been suggested to block the attack by antimicrobial peptides, although membrane disruption may be costly to combat through bacterial mutation [4, 11].

It has been proposed that host antimicrobial peptides have remained effective against invading pathogen through a process of co-evolution [37]. The lack of inherent or acquired resistance of gram-negative (this study) and gram-positive bacteria [12] and the ability of DGL13K to overcome bacterial defense mechanisms (proteolysis) that affect LGL13K give hope that antimicrobial peptides can be designed to address bacterial resistance without “arming the enemy”.

## Abbreviations

AMP: antimicrobial peptide
CFU: colony-forming units
ESBL: extended-spectrum beta-lactamase
KPC: Klebsiella Pneumoniae Carbapenemases
MDR: multidrug-resistant
MIC: minimal inhibitory concentration
PBS: phosphate-buffered saline
XDR: extensively-drug resistant

## References

[1] Hale JD, Hancock RE. Alternative mechanisms of action of cationic antimicrobial peptides on bacteria. Expert Rev Anti Infect Ther. 2007;5: 951–959. doi: 10.1586/14787210.5.6.951 18039080

[2] Lazar V, Martins A, Spohn R, Daruka L, Grezal G, Fekete G, et al. Antibiotic-resistant bacteria show widespread collateral sensitivity to antimicrobial peptides. Nat Microbiol. 2018;3: 718–731. doi: 10.1038/s41564-018-0164-0 29795541 PMC6544545

[3] Mangoni ML, McDermott AM, Zasloff M. Antimicrobial peptides and wound healing: biological and therapeutic considerations. Exp Dermatol. 2016;25: 167–173. doi: 10.1111/exd.12929 26738772 PMC4789108

[4] Zasloff M. Antimicrobial peptides of multicellular organisms. Nature. 2002;415: 389–395. doi: 10.1038/415389a 11807545

[5] Martinez B, Rodriguez A. Antimicrobial susceptibility of nisin resistant *Listeria monocytogenes* of dairy origin. FEMS Microbiol Lett. 2005;252: 67–72. doi: 10.1016/j.femsle.2005.08.025 16165322

[6] Habets MG, Brockhurst MA. Therapeutic antimicrobial peptides may compromise natural immunity. Biol Lett. 2012;8: 416–418. doi: 10.1098/rsbl.2011.1203 22279153 PMC3367763

[7] Perron GG, Zasloff M, Bell G. Experimental evolution of resistance to an antimicrobial peptide. Proc Biol Sci. 2006;273: 251–256. doi: 10.1098/rspb.2005.3301 16555795 PMC1560030

[8] Bell G, Gouyon PH. Arming the enemy: the evolution of resistance to self-proteins. Microbiology (Reading). 2003;149: 1367–1375. doi: 10.1099/mic.0.26265-0 . 12777478

[9] Dobson AJ, Purves J, Rolff J. Increased survival of experimentally evolved antimicrobial peptide-resistant *Staphylococcus aureus* in an animal host. Evol Appl. 2014;7: 905–912. doi: 10.1111/eva.12184 25469169 PMC4211720

[10] Fleitas O, Franco OL. Induced bacterial cross-resistance toward host antimicrobial peptides: a worrying phenomenon. Front Microbiol. 2016;7: 381. doi: 10.3389/fmicb.2016.00381 27047486 PMC4806371

[11] Bechinger B, Gorr SU. Antimicrobial peptides: mechanisms of action and resistance. J Dent Res. 2017;96: 254–260. doi: 10.1177/0022034516679973 27872334 PMC5298395

[12] Hirt H, Hall JW, Larson E, Gorr SU. A D-enantiomer of the antimicrobial peptide GL13K evades antimicrobial resistance in the Gram positive bacteria *Enterococcus faecalis* and *Streptococcus gordonii*. PLoS One. 2018;13: e0194900. doi: 10.1371/journal.pone.0194900 29566082 PMC5864073

[13] Abdolhosseini M, Sotsky JB, Shelar AP, Joyce PB, Gorr SU. Human parotid secretory protein is a lipopolysaccharide-binding protein: identification of an anti-inflammatory peptide domain. Mol Cell Biochem. 2012;359: 1–8. doi: 10.1007/s11010-011-0991-2 21833535 PMC3219827

[14] Geetha C, Venkatesh SG, Bingle L, Bingle CD, Gorr SU. Design and validation of anti-inflammatory peptides from human parotid secretory protein. J Dent Res. 2005;84: 149–153. doi: 10.1177/154405910508400208 15668332

[15] Gorr SU, Abdolhosseini M, Shelar A, Sotsky J. Dual host-defence functions of SPLUNC2/PSP and synthetic peptides derived from the protein. Biochem Soc Trans. 2011;39: 1028–1032. doi: 10.1042/BST0391028 21787342 PMC3417823

[16] Gorr SU, Sotsky JB, Shelar AP, Demuth DR. Design of bacteria-agglutinating peptides derived from parotid secretory protein, a member of the bactericidal/permeability increasing-like protein family. Peptides. 2008;29: 2118–2127. doi: 10.1016/j.peptides.2008.09.019 18952131

[17] Abdolhosseini M, Nandula SR, Song J, Hirt H, Gorr SU. Lysine substitutions convert a bacterial-agglutinating peptide into a bactericidal peptide that retains anti-lipopolysaccharide activity and low hemolytic activity. Peptides. 2012;35: 231–238. doi: 10.1016/j.peptides.2012.03.017 22484285 PMC3356437

[18] Hirt H, Gorr SU. Antimicrobial peptide GL13K is effective in reducing biofilms of *Pseudomonas aeruginosa*. Antimicrob Agents Chemother. 2013;57: 4903–4910. doi: 10.1128/AAC.00311-13 23917321 PMC3811403

[19] Gorr SU, Flory CM, Schumacher RJ. In vivo activity and low toxicity of the second-generation antimicrobial peptide DGL13K. PLoS One. 2019;14: e0216669. doi: 10.1371/journal.pone.0216669 31071184 PMC6508730

[20] Falciani C, Lozzi L, Pollini S, Luca V, Carnicelli V, Brunetti J, et al. Isomerization of an antimicrobial peptide broadens antimicrobial spectrum to gram-positive bacterial pathogens. PLoS One. 2012;7: e46259. doi: 10.1371/journal.pone.0046259 23056272 PMC3462775

[21] Hirsch EB, Raux BR, Zucchi PC, Kim Y, McCoy C, Kirby JE, et al. Activity of fosfomycin and comparison of several susceptibility testing methods against contemporary urine isolates. Int J Antimicrob Agents. 2015;46: 642–647. doi: 10.1016/j.ijantimicag.2015.08.012 26498988

[22] Magiorakos AP, Srinivasan A, Carey RB, Carmeli Y, Falagas ME, Giske CG, et al. Multidrug-resistant, extensively drug-resistant and pandrug-resistant bacteria: an international expert proposal for interim standard definitions for acquired resistance. Clin Microbiol Infect. 2012;18: 268–281. doi: 10.1111/j.1469-0691.2011.03570.x 21793988

[23] Clinical and Laboratory Standards Institute. Performance standards for antimicrobial susceptibility testing; 29^th^ informational supplement. Wayne, PA: Clinical and Laboratory Standards Institute; 2019: M100–S29. 10.1128/JCM.00213-21 PMC8601225 34550809

[24] Hancock REW. Modified MIC Method for Cationic Antimicrobial Peptides: University of British Columbia, British Columbia, Canada. 2001 [cited 2020 March]. Available from: http://www.cmdr.ubc.ca/bobh/methods.php;

[25] Chen A, Smith KP, Whitfield BA, Zucchi PC, Lasco TM, Bias TE, et al. Activity of minocycline against *Klebsiella pneumoniae* carbapenemase (KPC)-producing *Enterobacteriaceae* clinical isolates, with comparison to doxycycline and tigecycline. Diagn Microbiol Infect Dis. 2017;88: 365–367. doi: 10.1016/j.diagmicrobio.2017.05.004 28535946

[26] Moussa DG, Fok A, Aparicio C. Hydrophobic and antimicrobial dentin: A peptide-based 2-tier protective system for dental resin composite restorations. Acta Biomater. 2019;88:251–65. doi: 10.1016/j.actbio.2019.02.007 30753942 PMC6474255

[27] Wang D, Lin Z, Wang T, Ding X, Liu Y. An analogous wood barrel theory to explain the occurrence of hormesis: A case study of sulfonamides and erythromycin on *Escherichia coli* growth. PLoS One. 2017;12: e0181321. doi: 10.1371/journal.pone.0181321 28715457 PMC5513561

[28] Robinson GM, Tonks KM, Thorn RM, Reynolds DM. Application of bacterial bioluminescence to assess the efficacy of fast-acting biocides. Antimicrob Agents Chemother. 2011;55: 5214–5220. doi: 10.1128/AAC.00489-11 21876044 PMC3195015

[29] Butler DA, Biagi M, Tan X, Qasmieh S, Bulman ZP, Wenzler E. Multidrug resistant *Acinetobacter baumannii*: resistance by any other name would still be hard to treat. Curr Infect Dis Rep. 2019;21: 46. doi: 10.1007/s11908-019-0706-5 31734740

[30] Hirsch EB, Tam VH. Detection and treatment options for *Klebsiella pneumoniae* carbapenemases (KPCs): an emerging cause of multidrug-resistant infection. J Antimicrob Chemother. 2010;65: 1119–1125. doi: 10.1093/jac/dkq108 20378670

[31] Harmouche N, Aisenbrey C, Porcelli F, Xia Y, Nelson SED, Chen X, et al. Solution and solid-state nuclear magnetic resonance structural investigations of the antimicrobial designer peptide GL13K in membranes. Biochemistry (Mosc). 2017;56:4269–4278. doi: 10.1021/acs.biochem.7b00526 28699734

[32] Ye Z, Aparicio C. Interactions of two enantiomers of a designer antimicrobial peptide with structural components of the bacterial cell envelope. Journal of peptide science: an official publication of the European Peptide Society. 2022;28(1):e3299. Epub 2021/01/27. doi: 10.1002/psc.3299 33496073 PMC8310526

[33] Giacometti A, Cirioni O, Barchiesi F, Del Prete MS, Fortuna M, Caselli F, et al. In vitro susceptibility tests for cationic peptides: comparison of broth microdilution methods for bacteria that grow aerobically. Antimicrob Agents Chemother. 2000;44: 1694–1696. doi: 10.1128/AAC.44.6.1694-1696.2000 10817731 PMC89935

[34] Mattson MP. Hormesis defined. Ageing Res Rev. 2008;7: 1–7. doi: 10.1016/j.arr.2007.08.007 18162444 PMC2248601

[35] Stebbing ARD. Hormesis—The stimulation of growth by low levels of inhibitors. Sci Total Environ. 1982;22: 213–234. doi: 10.1016/0048-9697(82)90066-3 7043732

[36] Iavicoli I, Fontana L, Agathokleous E, Santocono C, Russo F, Vetrani I, et al. Hormetic dose responses induced by antibiotics in bacteria: A phantom menace to be thoroughly evaluated to address the environmental risk and tackle the antibiotic resistance phenomenon. Sci Total Environ. 2021;798:149255. doi: 10.1016/j.scitotenv.2021.149255 34340082

[37] Peschel A, Sahl HG. The co-evolution of host cationic antimicrobial peptides and microbial resistance. Nat Rev Microbiol. 2006;4: 529–536. doi: 10.1038/nrmicro1441 16778838

